# Comparative Accuracy of the InBios Scrub Typhus Detect IgM Rapid Test for the Detection of IgM Antibodies by Using Conventional Serology

**DOI:** 10.1128/CVI.00390-15

**Published:** 2015-09-23

**Authors:** Hugh W. F. Kingston, Stuart D. Blacksell, Ampai Tanganuchitcharnchai, Achara Laongnualpanich, Buddha Basnyat, Nicholas P. J. Day, Daniel H. Paris

**Affiliations:** a Mahidol-Oxford Tropical Medicine Research Unit, Faculty of Tropical Medicine, Mahidol University, Bangkok, Thailand; b Menzies School of Health Research, Charles Darwin University, Darwin, Northern Territory, Australia; c Centre for Tropical Medicine and Global Health, Nuffield Department of Clinical Medicine, Churchill Hospital, Headington, Oxford, United Kingdom; d Chiangrai Prachanukhru Hospital, Chiangrai, Thailand; e Oxford University Clinical Research Unit, Patan Academy of Health Sciences, Kathmandu, Nepal

## Abstract

This study investigated the comparative accuracy of a recombinant 56-kDa type-specific antigen-based rapid diagnostic test (RDT) for scrub typhus for the detection of IgM antibodies by using conventional serology in well-characterized serum samples from undifferentiated febrile illness patients. The RDT showed high specificity and promising comparative accuracy, with 82% sensitivity and 98% specificity for samples defined positive at an IgM indirect immunofluorescence assay positivity cutoff titer of ≥1:1,600 versus 92% and 95% at ≥1:6,400, respectively.

## TEXT

There is an urgent need for inexpensive, accurate point-of-care rapid diagnostic tests (RDTs) for scrub typhus. Clinical diagnosis on admission is rendered difficult by the nonspecificity of the presenting symptoms like fever and skin rash. The presence of an eschar at the mite inoculation site is a valuable diagnostic clue when found in combination with a positive RDT result, with positive and negative predictive values of 84.9 and 93.0%, respectively, but unfortunately, the varying occurrence of this lesion, especially in settings where scrub typhus is endemic, limits this approach (1). Currently, three modalities are used for the diagnosis of scrub typhus, i.e., culture, nucleic acid detection, and antibody detection. Culture of patient samples is insensitive, laborious, and expensive; nucleic acid detection is accurate in the early phase of infection, but its sensitivity falls with fever duration beyond 9 days (2). Antibody detection, traditionally by indirect immunofluorescence assay (IFA), requires skilled technicians and expensive equipment and is limited by the problem of background titers in settings where scrub typhus is endemic, antigen selection, and standardization (3). The rigorous use of paired serum samples with a ≥4-fold antibody titer rise required as a diagnostic endpoint has overcome some issues, but confounding factors include preexisting antibodies and cross-reactivity. Attempts to improve the gold standard have included combining all modalities into the scrub typhus infection criteria (STIC) proposed for diagnostic assay validations (2). However, the single admission endpoint titer conundrum is not yet adequately resolved. Recent Bayesian latent class modeling (LCM) data have highlighted the low specificity of admission and paired dynamic IFA IgM titers with low convalescent-phase titers, such as a ≥4-fold rise to ≤1:800 (1; Cherry Lim, personal communication).

An affordable, accurate point-of-care RDT that demonstrates a positivity cutoff at the population background antibody titer could potentially replace the admission IFA and impact patient management positively by guiding the administration of specific treatment. More data on the variation of background cutoff titers between geographical regions where scrub typhus is endemic are required, and more sensitive RDTs (i.e., RDTs that provide a positive result at a lower antibody titer) might be better in regions where scrub typhus is not endemic and less sensitive RDTs that are positive at higher cutoff titers are more useful in regions where scrub typhus is endemic. A comparative analysis of an RDT in a region where scrub typhus is endemic has shown improved specificity when using IgM over total antibody while maintaining sensitivity (4). IgM is produced immediately after pathogen exposure, with a shorter half-life in blood and lymphatics than more pathogen-specific IgG, which is produced later and provides a long-lasting response dependent on the previous exposure of the individual (5). Although IgG can persist for a long time and is thought to be more specific in paired samples, it can be associated with higher RDT false-positivity rates in areas where scrub typhus is endemic and the population is continuously exposed. Two important questions remain unresolved, (i) the longevity of IgM and IgG in human scrub typhus and (ii) which isotype appears earlier in naive and exposed populations. Nonhuman primate time course studies have shown that IgM and IgG can appear almost simultaneously in cynomolgus macaques (6, 7).

In this study, we evaluated a new commercial immunochromatographic assay-based RDT based on the Orientia tsutsugamushi strain Karp, Kato, Gilliam, and TA716 recombinant 56-kD type-specific antigen (Scrub Typhus Detect IgM rapid test; InBios International Inc., Seattle WA, USA). Two prototype RDT versions that use either a polyclonal antibody (PAb) or a monoclonal antibody (MAb) as the secondary antibody for IgM detection were tested.

The InBios RDTs were performed with the same batch and lot (800231 and NB273/52, respectively) by using serum samples (10 μl of serum per test strip) according to the manufacturer's instructions. Previously characterized admission serum samples (total *n* = 100) were used that were collected from febrile illness patients enrolled in ethically and fully Institutional Review Board-approved prospective “causes-of-fever” studies performed in Udon Thani in northeastern Thailand (2000 and 2001; *n* = 85) and Kathmandu, Nepal (2008 to 2011; *n* = 15) (8, 9). The samples included were from confirmed scrub typhus cases (*n* = 21) meeting any of the previously defined stringent STIC, i.e., culture positivity and/or an admission IgM antibody titer of >1:12,800 and/or a ≥4-fold rising IgM IFA antibody titer and/or positivity for two or more of three PCR gene targets. Murine typhus cases with paired dynamic serology and/or quantitative PCR positivity (*n* = 23) and dengue cases with NS1 antigen positivity (*n* = 5) were included. The other cases represented patients with undifferentiated febrile illness (*n* = 51) with negative scrub typhus, murine typhus, and NS1 antigen test results (2, 10).

**FIG 1:**
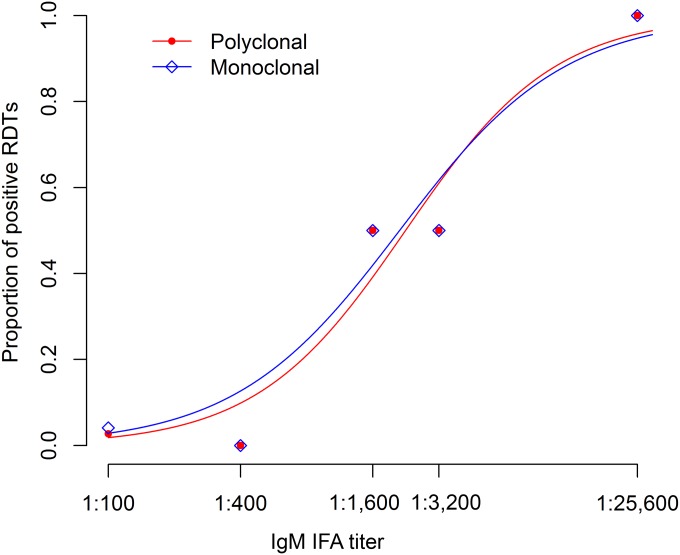
Relationship between IFA titers and RDT positivity. Shown are the proportions of positive RDTs at different IFA titers plotted on a logarithmic scale. Logistic regression was used to describe the sigmoidal relationship between RDT positivity and IFA titers, illustrating the low proportions of RDT positivity at IFA titers of <1:3,200. No significant difference between the two RDT versions was observed (secondary PAb detection, red; secondary MAb detection, blue).

The IFA used pooled O. tsutsugamushi Karp, Kato, and Gilliam antigens to detect IgM antibodies with IFA slides produced by the Australian Rickettsial Reference Laboratory (Geelong, Australia). Patient serum samples were serially 2-fold diluted from 1:100 to 1:25,600, and the endpoint was determined by two experienced staff members as the highest titer displaying specific fluorescence (11). Three independent laboratory technicians read the developed RDTs blinded to each other's results, and the majority interpretation was final. Sensitivity and specificity were calculated by using a range of IFA endpoint cutoff titers for positivity, and binomial 95% confidence intervals (CIs) were calculated. Kappa statistics were calculated for interreader variation. Statistical analysis and logistic regression (Fig. 1) were performed by using Stata/IC software (version 13.0; StataCorp, College Station, TX, USA) and plotted by using R version 3.1.1 (available at http://www.r-project.org).

The challenges of a point-of-care RDT are comparable to those of a single-titer admission IFA, albeit with a simplified procedure and a clear-cut endpoint. In this study, we did not attempt to estimate the classic diagnostic accuracy of the InBios RDT—that would require a prospective study design—but rather attempted to define the antibody titer associated with optimal RDT performance in a set of characterized samples as its comparative accuracy. Hence, we assessed the agreement of RDT positivity rates with a range of samples with predefined different IgM IFA admission titers. Table 1 summarizes the assay results and the respective sensitivity and specificity values at the different IFA cutoff titers.

**TABLE 1:** Summary of characterized patient admission serum samples and RDT performance in this study^*a*^

IFA reciprocal titer cutoff (no. of samples/100 with IFA IgM titer cutoff) and RDT version	No. of RDT results^*b*^				% Sensitivity (95% CI)	% Specificity (95% CI)
	TP	FP	FN	TN		
≥400 (27)						
PAb	14	2	13	71	52 (32–71)	97 (90–100)
MAb	14	3	13	70	52 (32–71)	96 (88–99)
≥1,600 (17)						
PAb	14	2	3	81	82 (57–96)	98 (92–100)
MAb	14	3	3	80	82 (57–96)	96 (90–99)
≥6,400 (13)						
PAb	12	4	1	83	92 (64–100)	95 (89–99)
MAb	12	5	1	82	92 (64–100)	94 (87–98)
≥25,600 (11)						
PAb	11	5	0	84	100 (72–100)	94 (87–98)
MAb	11	6	0	83	100 (72–100)	93 (86–97)

a The results shown are stratified by IFA IgM antibody positivity titers (horizontal rows) with corresponding InBios RDT diagnostic accuracies reported separately for IgM detection modalities, PAb versus MAb. Although the number of characterized samples with confirmed scrub typhus was 21/100, more samples had low IFA IgM positivity, i.e., of all of the samples, if an IFA cutoff titer of ≥1:400 was chosen, then 14 of these were true positives and 13 were false negatives—with rising IFA IgM titers, the rate of false negativity decreased.

b RDT results: TP, true positive; FP, false positive; FN, false negative; TN, true negative.

The proportion of RDTs with a positive result at each IFA IgM titer increased with higher IFA IgM titers (test for trend; *P* < 0.001). All (100%) of the RDTs provided negative results at IFA titers of ≤1:400, and all (100%) of the RDTs provided positive results at titers of ≥1:25,600. The results show that both versions of the new test identify the same number of positive samples identified by IFA when the reciprocal antibody cutoff titer is high, thereby giving 100% sensitivity. Although the tests agree well if the sample has a high antibody titer, the new test misses some of the IFA-positive samples at low cutoff titers, with a subsequent reduction in sensitivity. Plotting the proportion of positive RDTs at different IFA titers delineates a sigmoidal relationship, with increasing proportions of RDT positivity at titers of ≥1:3,200 (Fig. 1). This response was comparable to that of the previously assessed PanBio RDT (12). There was a minimal difference between the two secondary-antibody versions in terms of the proportions positive at the different IFA titers. The interpretation of bands was perceived to be more difficult because of weaker, paler, and more smeared bands in positive samples when using RDTs based on MAbs versus RDTs based on PAbs, which was reflected by a marginally higher kappa value of 0.97 of the high antigen density version than the 0.93 of the low-density version, respectively (data not shown).

The RDTs assessed in this study were specific and sensitive for the detection of high IFA titer samples only. This RDT would therefore be expected to perform well in areas where scrub typhus is endemic, where a higher background antibody titer would be expected in the population, as low titers would result in a negative RDT result and higher titers would be detected with good diagnostic accuracy.

It is noteworthy that a >64-fold difference in IgM antibody concentrations exists between the samples with titers of 1:400 and >1:25,600. In samples with a low IFA titer (≤1:400), the RDT results were generally negative, contributing to a high specificity. It is not known if the antibodies in serum samples with an IFA titer of 1:400 are different from the antibodies in samples with a 1:25,600 titer in either affinity or target. However, we have shown in recent Bayesian LCM analyses that paired dynamic IFA IgM titers with low convalescent-phase titers, such as a ≥4-fold rise to ≤1:800, contribute to the low specificity of the IFA assay, and therefore a higher endpoint cutoff positivity titer needs to be considered (1; Lim, personal communication).

The choice of an IFA positivity cutoff endpoint titer of 1:400 over 1:1,600 to 1:6,400 results in a stepwise improvement of the InBios RDT's diagnostic accuracy, with sensitivities (95% CIs) ranging from 52% (32 to 71%) over 82% (57 to 96%) to 92% (64 to 100%) while retaining a specificity of ≥94% (Table 1). The size of the study data set did not allow for in-depth and detailed analyses; however, it can be safely assumed that the positivity cutoff titer of the InBios RDT lies around the 1:1,600-to-1:3,200 titer range.

The RDT under evaluation may have benefited from the inclusion of antigenically disparate recombinant immunodominant 56-kDa antigens from four O. tsutsugamushi strains, three more than the PanBio IgM RDT and one more than the reference IFA, the additional O. tsutsugamushi TA716 strain. A study limitation is that the benefit of a broader antigen spectrum covered by the RDT would have gone unnoticed in the present evaluation, as anti-TA716 antibodies would not have been detected by the IFA used (based on the Karp, Gilliam, and Kato strains), which would have increased the RDT's false-positivity rate because of false-negative IFA results.

In conclusion, the InBios RDTs tested here show promising performance characteristics for use in zones where scrub typhus is endemic, where the admission IgM IFA positivity cutoff titer would lie around 1:1,600 to 1:3,200. The RDT assay based on endpoint detection with a PAb is preferable for a prospective evaluation. Attention should be given to understanding why the RDTs are negative at a cutoff of ≤1:400 and how this can be improved to develop tests for use where scrub typhus is not endemic.
